# Bioenergetic imbalance and oxidative stress in the pathophysiology of septic encephalopathy

**DOI:** 10.1186/cc11960

**Published:** 2013-03-19

**Authors:** J D'Avila, R Rodrigues, H Castro-Faria-Neto, M Oliveira, F Bozza

**Affiliations:** 1 Oswaldo Cruz Foundation - FIOCRUZ, Rio de Janeiro, Brazil; 2 Federal University of Rio de Janeiro (UFRJ) and D'Or Institute for Research and Education (IDOR), Rio de Janeiro, Brazil; 3 UFRJ, Rio de Janeiro, Brazil; 4 Oswaldo Cruz Foundation-FIOCRUZ and IDOR, Rio de Janeiro, Brazil

## Introduction

Septic encephalopathy is a frequent complication in severe sepsis but its pathogenesis and mechanisms are not fully understood. Oxygen supply and utilization are critical for organ function, especially for the brain, a tissue extremely dependent on oxygen and glucose. Disturbances in oxygen utilization are common in sepsis and a number of mitochondrial dysfunctions have been described in different tissues in septic animals as well as in septic patients. Our group described mitochondrial dysfunctions in the brain during experimental sepsis.

## Methods

Experimental sepsis was induced by endotoxemia (LPS 10 mg/ kg i.p.) in Sprague-Dawley rats and by polymicrobial fecal peritonitis in Swiss mice. Brain glucose uptake was observed *in vivo *in endotoxemic rats using positron emission tomography with [^18^F] fiuorodeoxyglucose and autoradiography with 2-deoxy-^14^C-glucose.

## Results

Mice with polymicrobial sepsis present hypoglycemia, hyperlactatemia and long-term cognitive impairment. We observed a rapid increase in the uptake of fluorescent glucose analog 2-deoxy-2-((7-nitro-2,1,3-benzoxadiazol-4-yl)amino)-D-glucose in brain slices from septic mice *in vitro*. A similar increase in brain glucose uptake was observed *in vivo *in endotoxemic rats. Remarkably, the increase in glucose uptake started 2 hours after LPS injection, earlier than other organs. The brains of mice with experimental sepsis presented neuroinflammation, mitochondrial dysfunctions and oxidative stress, but mitochondria isolated from septic brains generated less ROS *in vitro *in the first 24 hours. This led us to investigate the role of NADPH oxidase, an enzyme induced during innate immune response, as a potential source of reactive oxygen species in experimental sepsis. Inhibiting NADPH oxidase with apocynin acutely after sepsis prevented cognitive impairment in mice.

## Conclusion

Our data indicate that a bioenergetic imbalance and oxidative stress is associated with the pathophysiology of septic encephalopathy. We are observing a new metabolic phenotype in the brain during sepsis, characterized by a rapid increase in glucose uptake and mitochondrial dysfunctions that may be secondary to inflammation and hypoxia.

